# Supplementary material for: Psilocin fosters neuroplasticity in iPSC-derived human cortical neurons
Source: eLife. 2026 Mar 27;14:RP104006. doi: 10.7554/eLife.104006 (PMC13030890; doi:10.7554/eLife.104006)
Supplement: Figure 1—figure supplement 1—source data 1. [file elife-104006-fig1-figsupp1-data1.zip › Figure 1-figure supplement 1C_uncropped_labelled.pptx]

## Slide 1
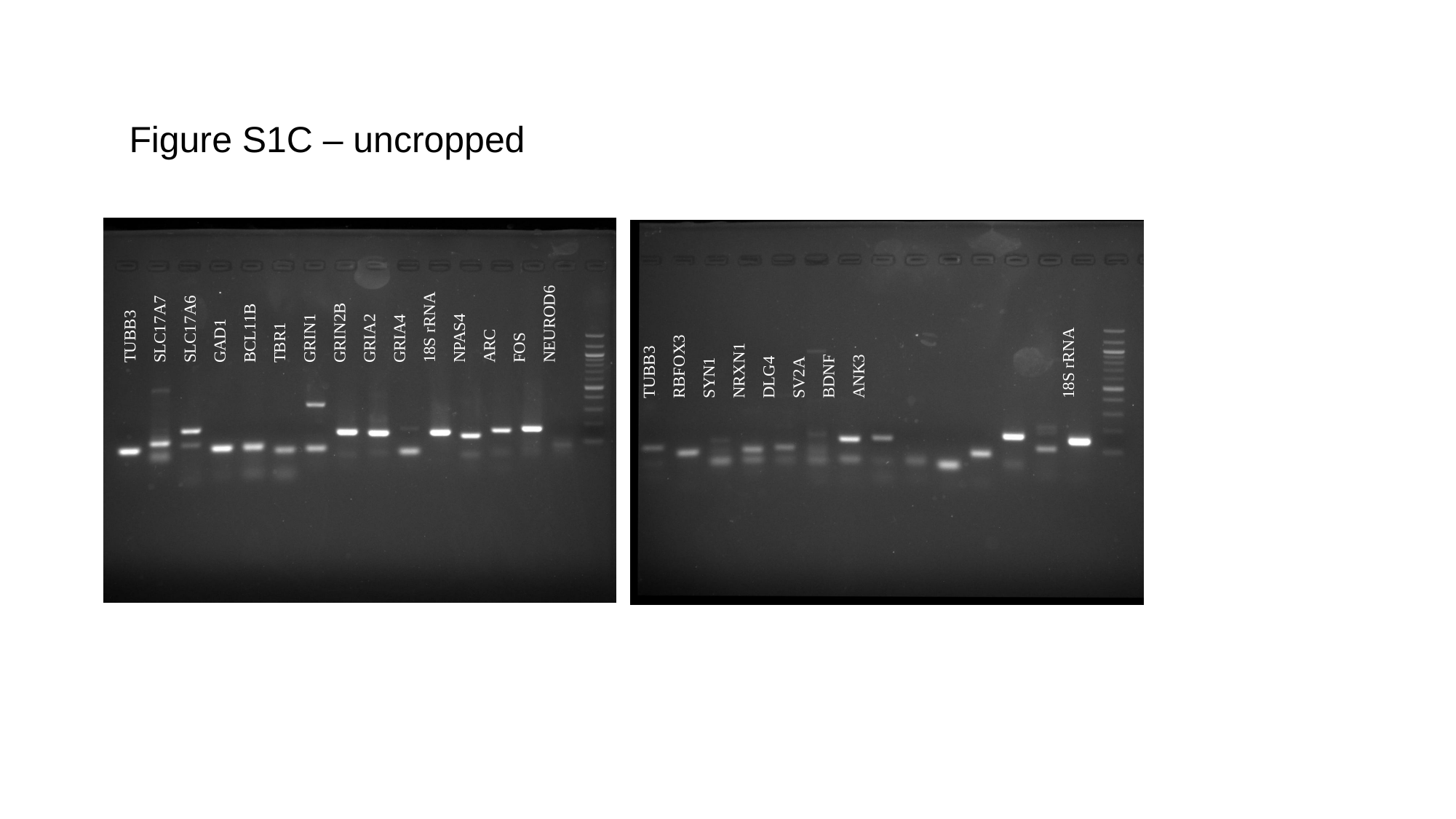

Figure S1C – uncropped
TUBB3
SLC17A7
SLC17A6
GAD1
BCL11B
TBR1
GRIN1
GRIN2B
GRIA2
GRIA4
18S rRNA
NPAS4
ARC
FOS
NEUROD6
TUBB3
RBFOX3
SYN1
NRXN1
DLG4
SV2A
BDNF
ANK3
18S rRNA
