# Supplementary figures and images for: Psilocin fosters neuroplasticity in iPSC-derived human cortical neurons
Source: eLife. 2026 Mar 27;14:RP104006. doi: 10.7554/eLife.104006 (PMC13030890; doi:10.7554/eLife.104006)

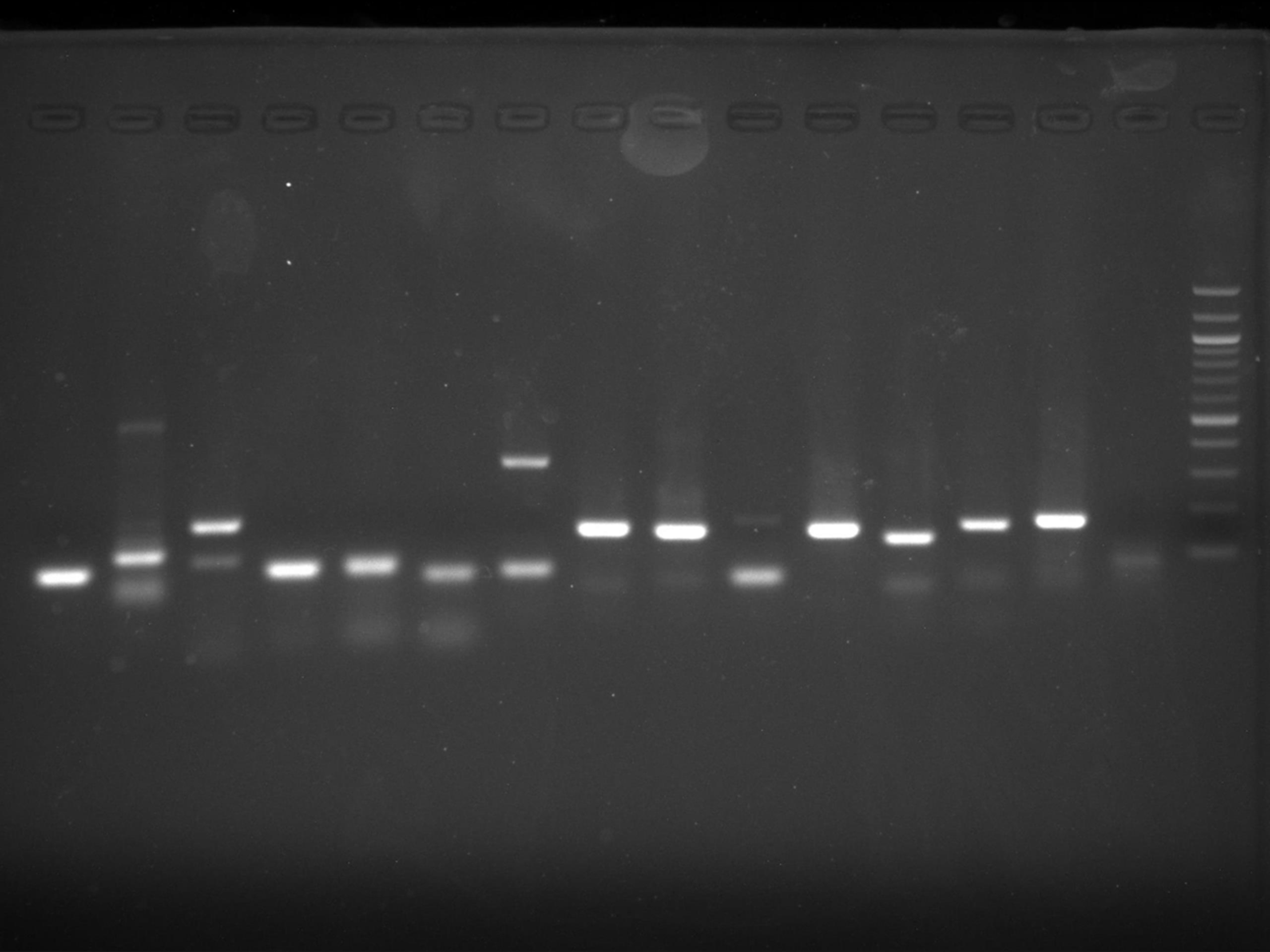

Supplement: Figure 1—figure supplement 1—source data 2. [file elife-104006-fig1-figsupp1-data2.zip › Figure 1-figure supplement 1C_1.tiff]

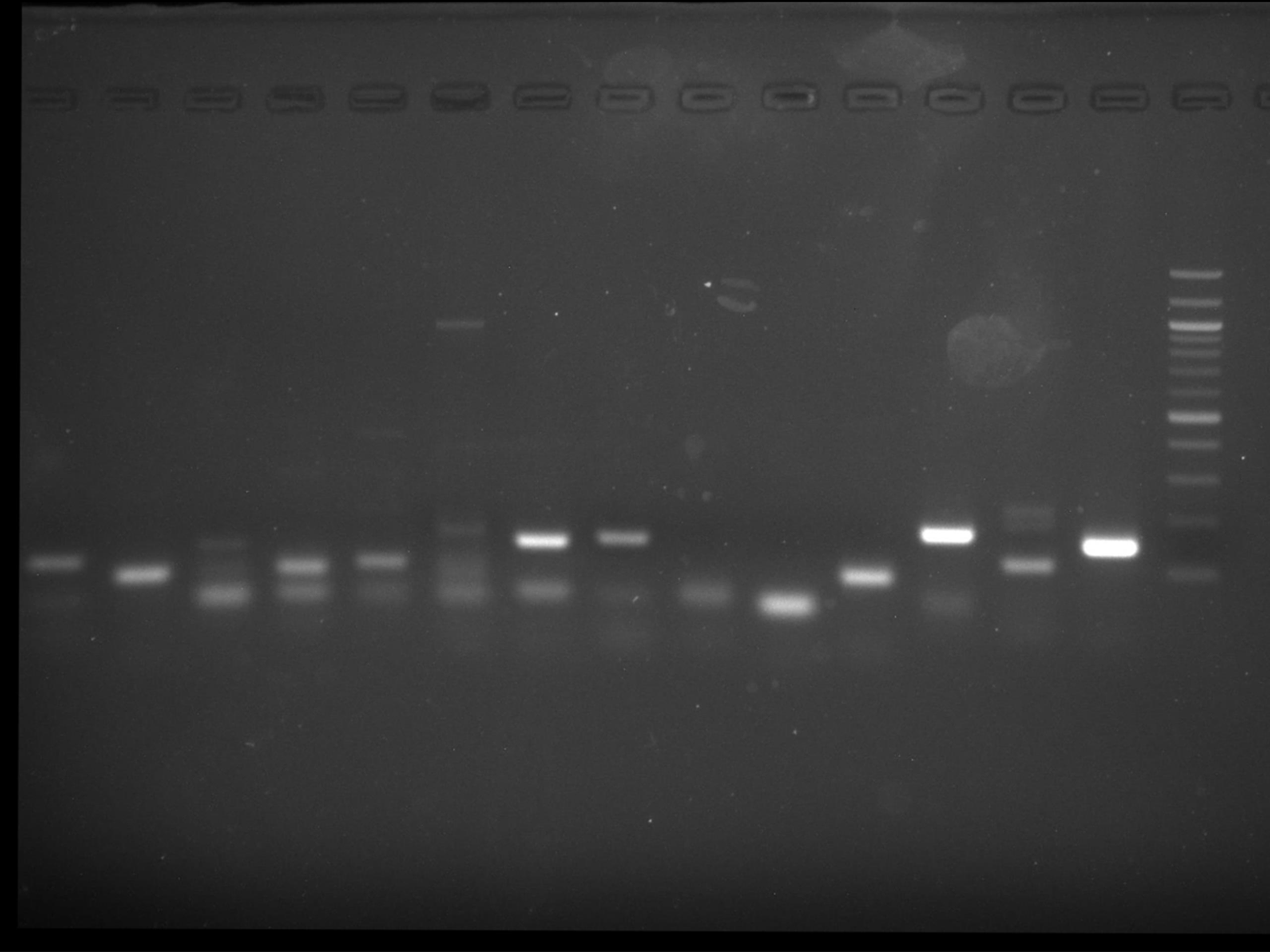

Supplement: Figure 1—figure supplement 1—source data 2. [file elife-104006-fig1-figsupp1-data2.zip › Figure 1-figure supplement 1C_2.tiff]

## Slide 1
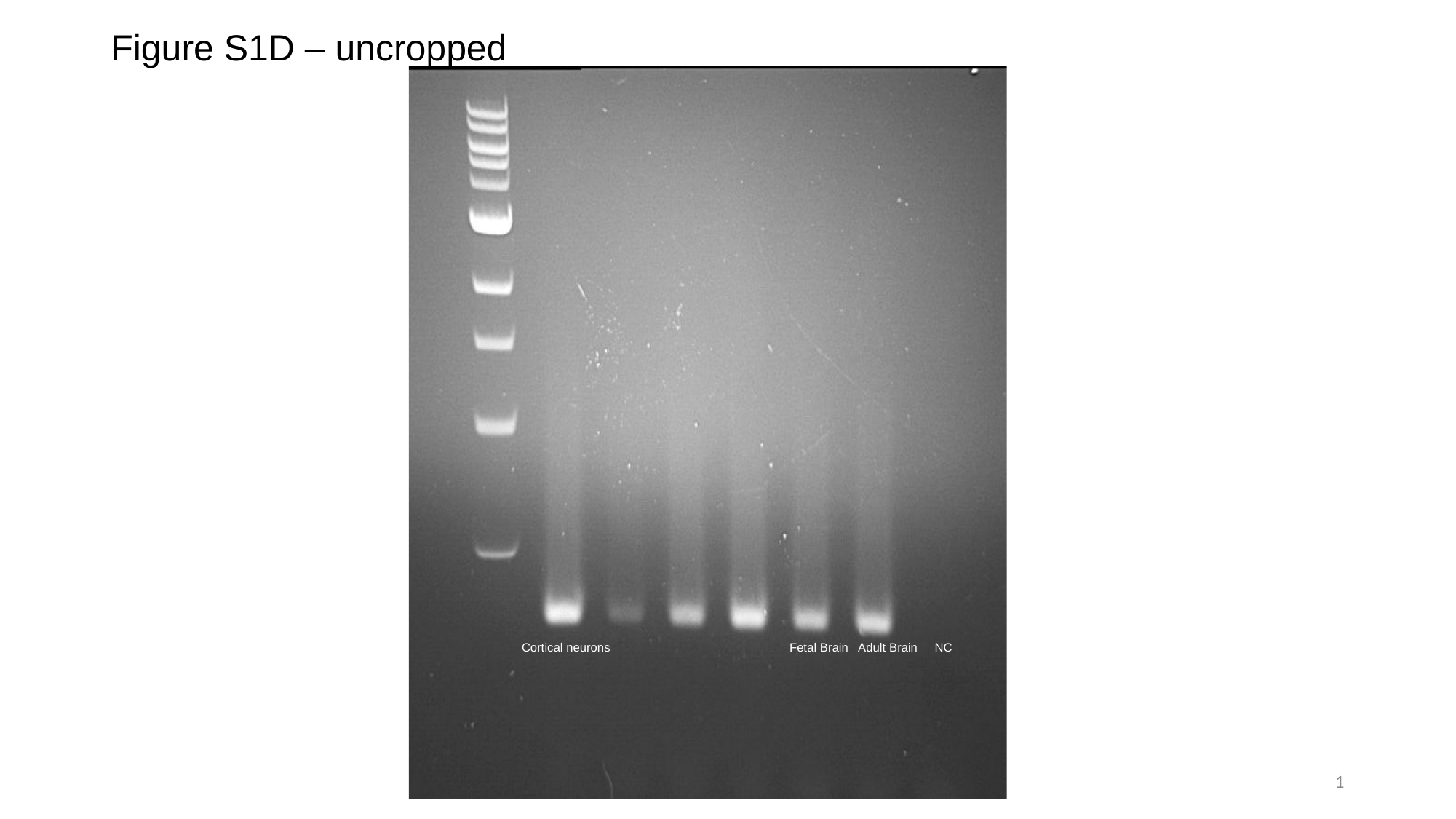

Figure S1D – uncropped
Cortical neurons Fetal Brain Adult Brain NC
1

Supplement: Figure 1—figure supplement 1—source data 3. [file elife-104006-fig1-figsupp1-data3.zip › Source File Figure 1-figure supplement 1D_uncropped_labelled.pptx]

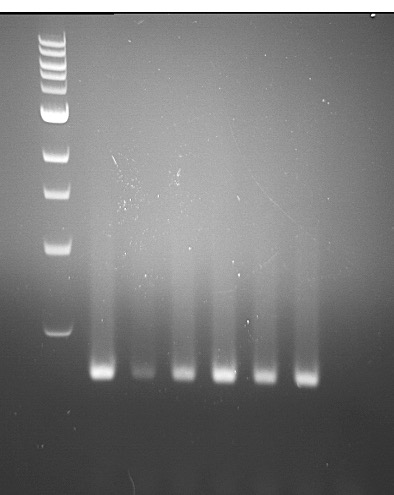

Supplement: Figure 1—figure supplement 1—source data 4. [file elife-104006-fig1-figsupp1-data4.zip › Source File Figure 1-figure supplement 1D_uncropped.jpg]

## Slide 1
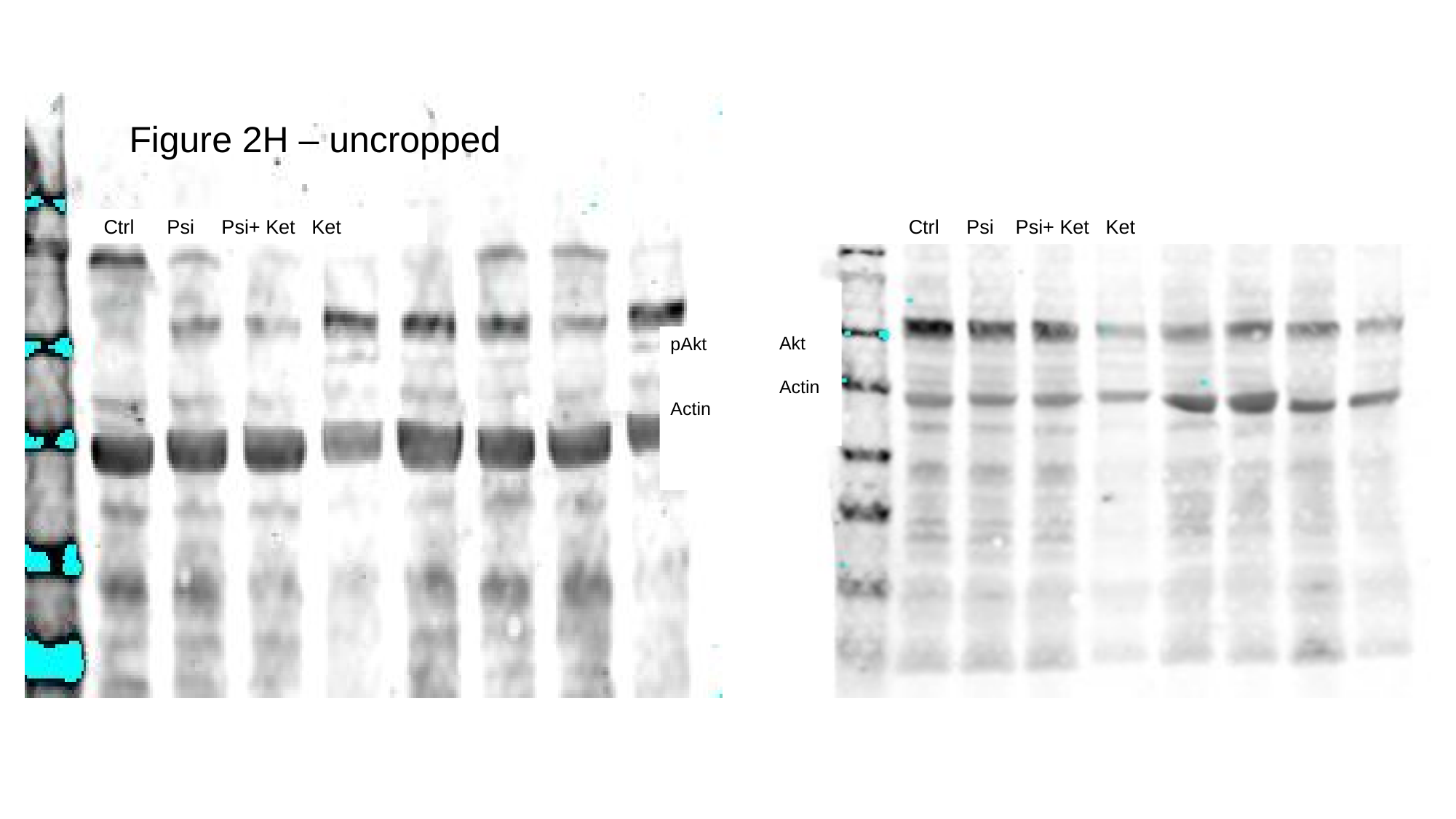

Figure 2H – uncropped
 Ctrl Psi Psi+ Ket Ket
 Ctrl Psi Psi+ Ket Ket
Akt
Actin
pAkt
Actin

Supplement: Figure 2—source data 1. [file elife-104006-fig2-data1.zip › Source Data Fig. 2H_uncropped_labelled.pptx]

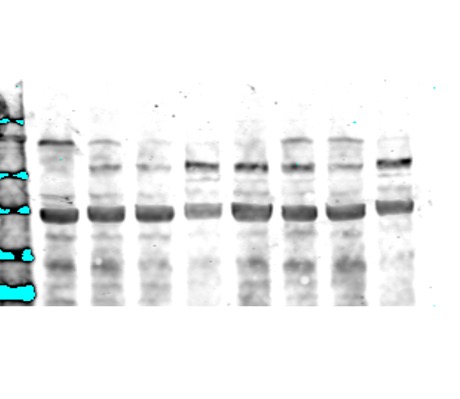

Supplement: Figure 2—source data 2. [file elife-104006-fig2-data2.zip › Source Data Fig. 2H_1.jpg]

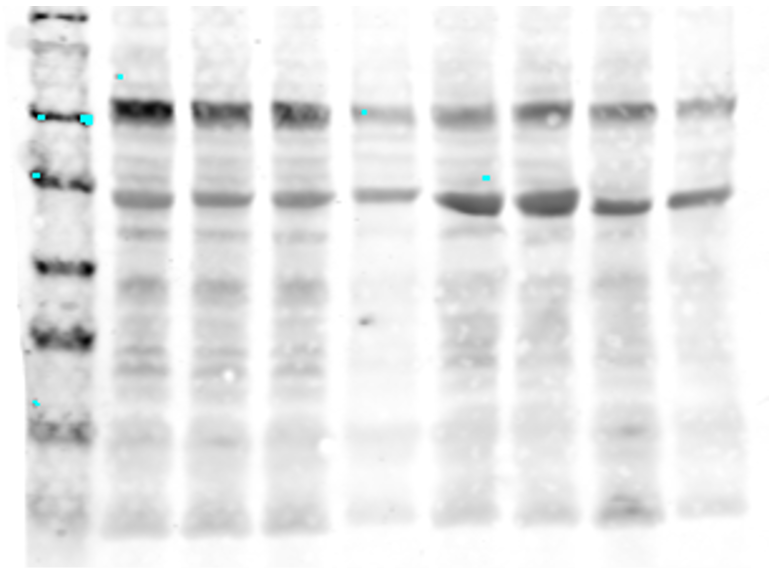

Supplement: Figure 2—source data 2. [file elife-104006-fig2-data2.zip › Source Data Fig. 2H_2.png]

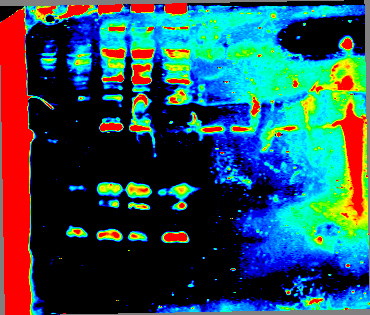

Supplement: Figure 2—figure supplement 1—source data 2. [file elife-104006-fig2-figsupp1-data2.zip › Figure S2H_uncropped/Source File Fig. 2H_3.tif]

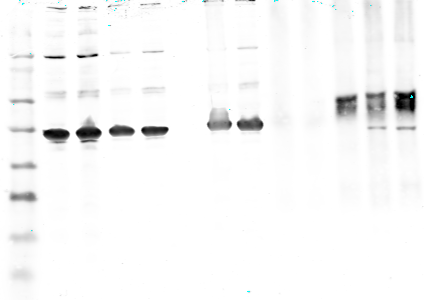

Supplement: Figure 2—figure supplement 1—source data 2. [file elife-104006-fig2-figsupp1-data2.zip › Figure S2H_uncropped/Source File Fig. 2H_2.tif]

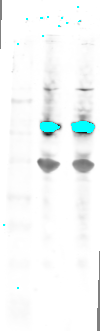

Supplement: Figure 2—figure supplement 1—source data 2. [file elife-104006-fig2-figsupp1-data2.zip › Figure S2H_uncropped/Source File Fig. 2H_1.tif]

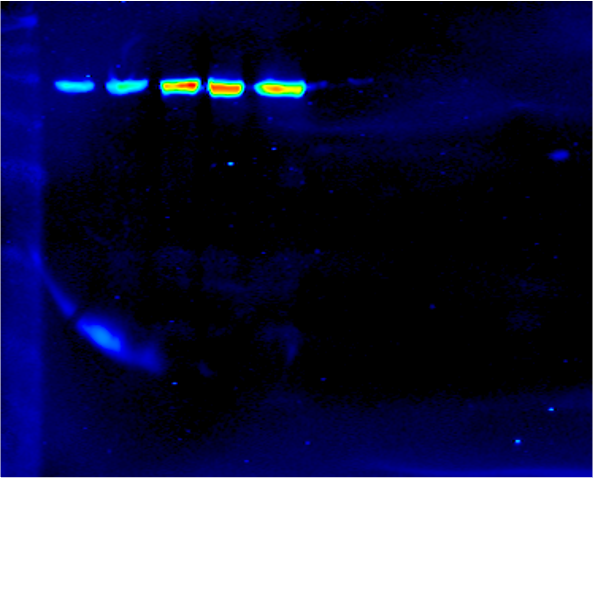

Supplement: Figure 2—figure supplement 1—source data 2. [file elife-104006-fig2-figsupp1-data2.zip › Figure S2H_uncropped/Source File Fig. 2H_4.png]

## Slide 1
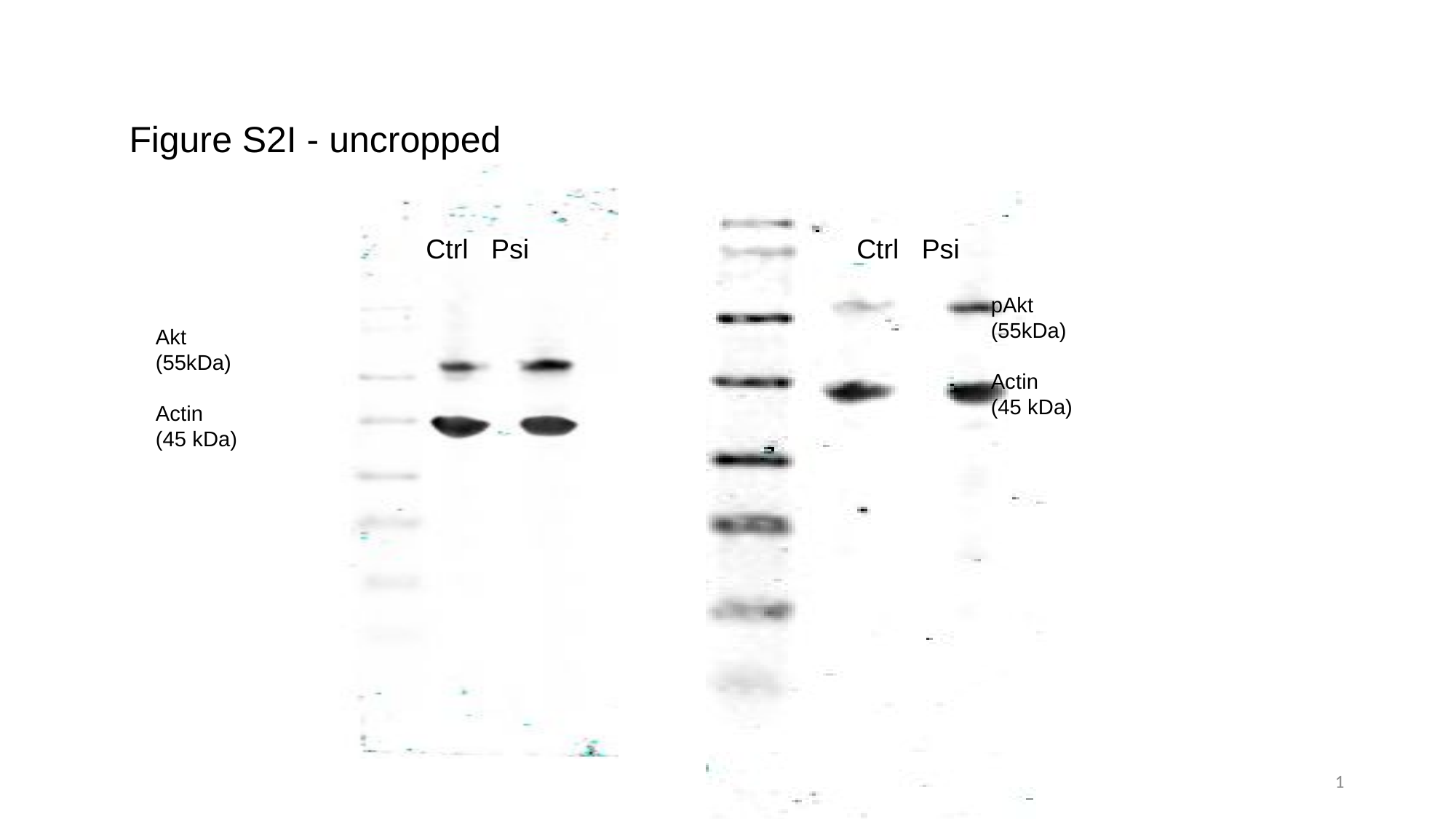

Figure S2I - uncropped
Ctrl Psi
Ctrl Psi
pAkt
(55kDa)
Actin
(45 kDa)
Akt
(55kDa)
Actin
(45 kDa)
1

Supplement: Figure 2—figure supplement 1—source data 3. [file elife-104006-fig2-figsupp1-data3.zip › Figure S2I_uncropped_labelled.pptx]

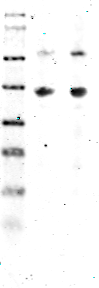

Supplement: Figure 2—figure supplement 1—source data 4. [file elife-104006-fig2-figsupp1-data4.zip › Source Data Fig. 2-figure supplement 1I_1.tif]

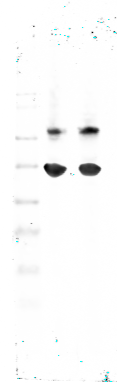

Supplement: Figure 2—figure supplement 1—source data 4. [file elife-104006-fig2-figsupp1-data4.zip › Source Data Fig. 2-figure supplement 1I_2.tif]
