# Supplementary material for: Psilocin fosters neuroplasticity in iPSC-derived human cortical neurons
Source: eLife. 2026 Mar 27;14:RP104006. doi: 10.7554/eLife.104006 (PMC13030890; doi:10.7554/eLife.104006)
Supplement: Figure 2—figure supplement 1—source data 1. [file elife-104006-fig2-figsupp1-data1.zip › Figure S2H_uncropped_labelled.pptx]

## Slide 1
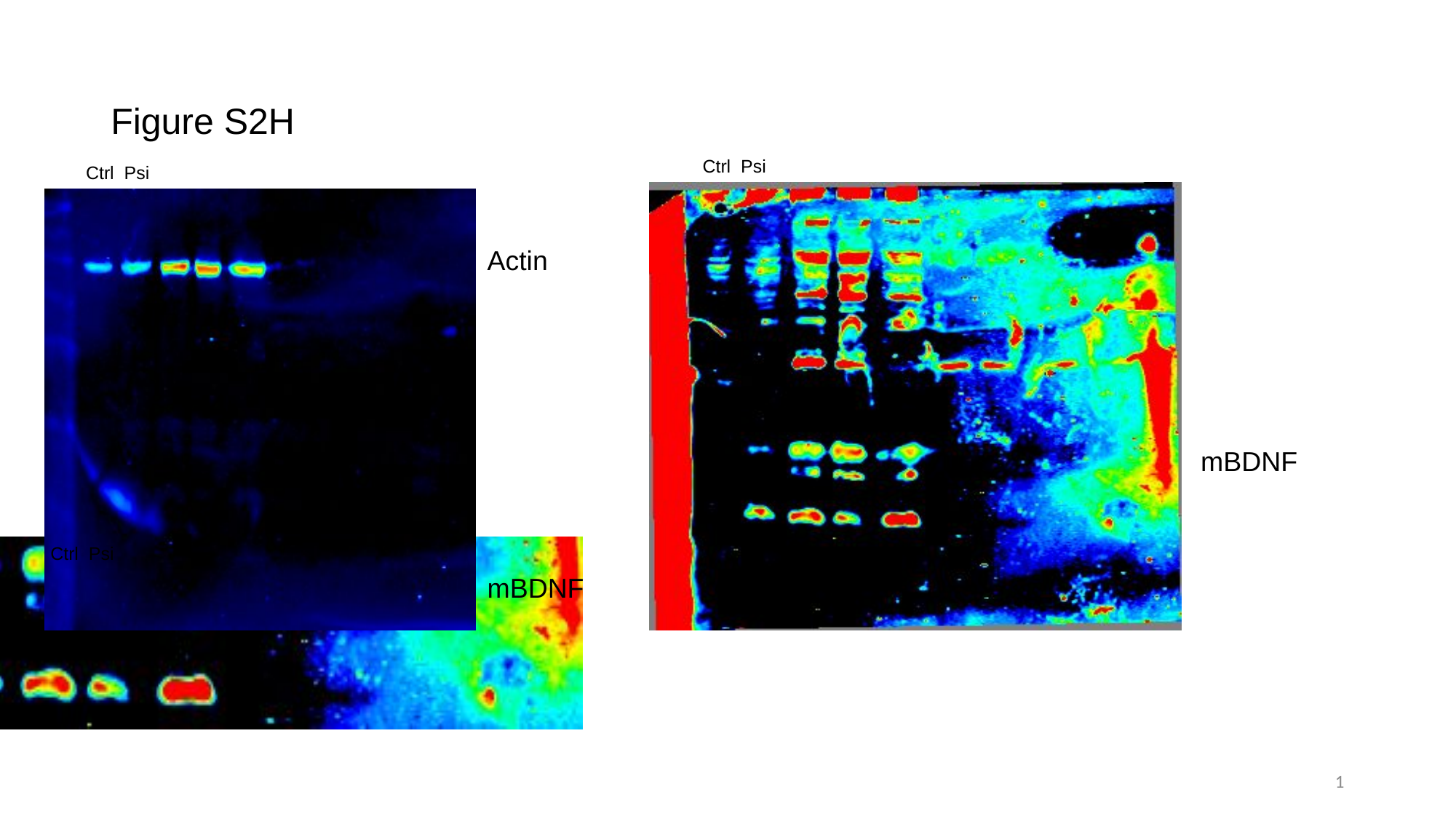

# Figure S2H
mBDNF
Ctrl Psi
Ctrl Psi
Actin
mBDNF
Ctrl Psi
1

## Slide 2
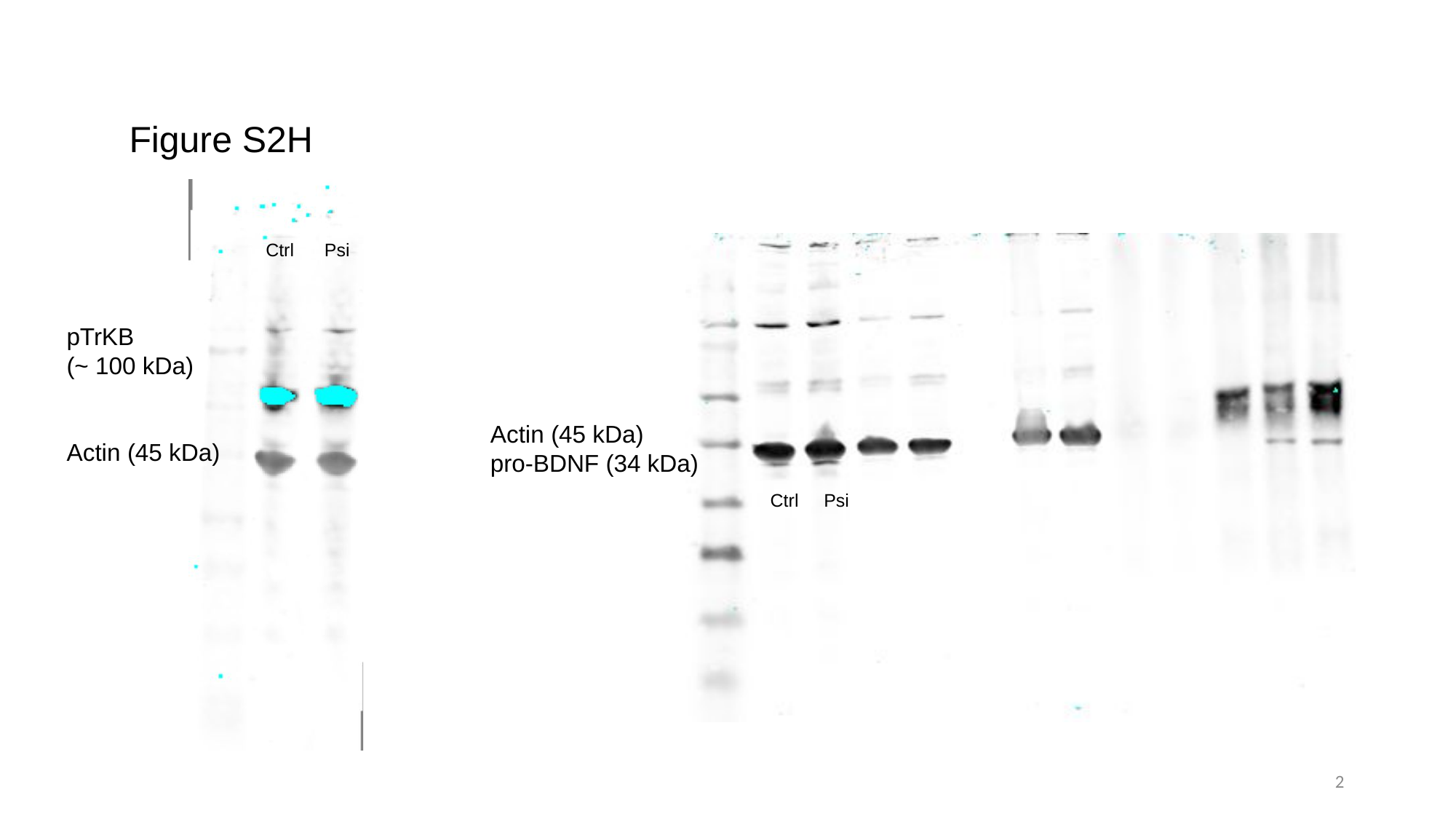

Figure S2H
Ctrl Psi
pTrKB
(~ 100 kDa)
Actin (45 kDa)
Actin (45 kDa)
pro-BDNF (34 kDa)
Ctrl Psi
2
