# Supplementary material for: Psilocin fosters neuroplasticity in iPSC-derived human cortical neurons
Source: eLife. 2026 Mar 27;14:RP104006. doi: 10.7554/eLife.104006 (PMC13030890; doi:10.7554/eLife.104006)
Supplement: Supplementary file 1. [file elife-104006-supp1.docx]

**Supplementary**

Supplementary file 1a: ALU insertions primers based on^55^.

| \| ALU insertions \| Primers \| \| --- \| --- \| \| AL-1a \| GCAGATTTCAGGTCATTATTG/ GGTCACTAGAGACCGTATCTGTA \| \| Al-2a \| TGTTTAAAATGATCAAAATTAGG/ AGATCTCTGGCTAAGTTCTTTAC \| \| Al-3a \| ACCAAATACCCCTCAGAAGA/ CCTGACCTAGTGTACCCATTAG \| \| Al-4a \| GAGGTTAGACAACATACATTATATCA/ CCCTATTGTTTACATCTAATTCTA \| \| Al-5a \| ACGCAAGGACTTAATTGTAAC/ TAATGTTTGGATTATTCCTGGA \| \| Al-6a \| AGCTGATATATCTCAATAAGCAAC/ GATTAAAATCACTTGTCTGAGACT \| \| Al-7a \| CAGAGTAAAGCTACAGTAAGACAGTA/ ATAACATAGGGAAAATCACTTCTA \| \| Al-8a \| TGTCTGTTTCAGCTCTTCACTA/ ATACTATTTGGTTGACCCTACTG \| \| Al-9a \| TTGGAACCTTTGGTAAGTAACT/ AAAGCCACAGAAATACATCTAAT \| \| Al-10 \| TGCCAAACAGTGATTATAGAAC/ CTGTCTAGAAGACAACTAGGAATAG \| \| Al-11 \| CTATGATGTGCTATGGAAGGTAGGT/ CCTAGTCCTCATAAATTAGTAACCTG \| \| Al-12 \| ATTTGGGAGTTTCCATTATGA/ TATTAACCATATTATGTGCCAAGA \| |
| --- | --- | --- | --- | --- | --- | --- | --- | --- | --- | --- | --- | --- | --- | --- | --- | --- | --- | --- | --- | --- | --- | --- | --- | --- | --- | --- |

Supplementary file 1b: Primary antibodies used for immunocytochemistry (ICC).

| Antibody (clone) | Host species | Dilution | Blocking Solution in 10% Fetal bovine serum  (FBS) | Manufacturer | Catalogue number | | Registered office |
| --- | --- | --- | --- | --- | --- | --- | --- |
| BDNF | rabbit | 1:300 | 0.1% Saponin | Elabscience | E-AB-18244.200 | Houston, USA | |
| CTIP-2 (25B6) | rat | 1:500 | 0.3% Triton X | Abcam | ab18465 | Cambridge, UK | |
| cFOS (9F6) | rabbit | 1:500 | 0.3% Triton X | Cell Signaling Technologies | 2250 | Danvers, USA | |
| FOXA2 | goat | 1:500 | 0.3% Triton X | R&D Systems | AF2400 | Minneapolis, USA | |
| OCT3/4 (A-9) | mouse | 1:500 | 0.3% Triton X | Santa Cruz | Sc-365509 | Santa Cruz, USA | |
| GFAP | mouse | 1:500 | 0.1% Triton X | Synaptic Systems | 173011 | Göttingen, Germany | |
|  |  |  |  |  |  |  | |
| MAP2 | chicken | 1:7000 | 0.1% Saponin/ 0.1% - 0.3% Triton X | BioLegend | 822501 | San Diego, USA | |
| mCherry | rabbit | 1:1000 | 0.3% Triton X | Cell Signaling Technologies | 43590S | Danvers, USA | |
| NESTIN  196908 | mouse | 1:600 | 0.3% Triton X | R&D Systems | MAB-1259 | Minneapolis, USA | |
| NeuN (D4G4O) | rabbit | 1:200 | 0.3% Triton X | Cell Signaling Technologies | 24307S | Danvers, USA | |
| PAX6 (Poly19013) | rabbit | 1:500 | 0.3% Triton X | Biolegend | 901301 | San Diego, USA | |
| PSD-95 | rabbit | 1:250 | 0.1% Saponin | Cell Signaling Technologies | 2507S | Danvers, USA | |
| SOX2 (D6D9) | rabbit | 1:500 | 0.3% Triton X | Cell Signaling Technologies | 3579S | Danvers, USA | |
| Synapsin I/II/III (A17080A) | mouse | 1:500 | 0.1% Saponin | BioLegend | 853701 | San Diego, USA | |
| TAU | guinea-pig | 1:700 | 0.1% Saponin/ 0.1% - 0.3% Triton X | Synaptic Systems | 314004 | Göttingen, Germany | |
| TBR1 | rabbit | 1:500 | 0.3% Triton X | Abcam | ab31940 | Cambridge, UK | |
| vGLUT2 | guinea-pig | 1:200 | 0.1% Triton X | Synaptic Systems | 135304 | Göttingen, Germany | |

Supplementary file 1c: Secondary antibodies used for ICC.

| Antibody | Host species | Manufacturer | Catalogue number | Registered office |
| --- | --- | --- | --- | --- |
| anti-chicken IgG  Alexa Fluor-488 | goat | Thermo Fisher Scientific | A11039 | Waltham, USA |
| anti-guinea-pig IgG Alexa Fluor-488 | goat | Thermo Fisher Scientific | A11073 | Waltham, USA |
| anti-guinea-pig IgG Alexa Fluor-568 | goat | Thermo Fisher Scientific | A11075 | Waltham, USA |
| anti-mouse IgG Alexa Fluor-488 | goat | Thermo Fisher Scientific | A11001 | Waltham, USA |
| anti-mouse IgG Alexa Fluor-568 | goat | Thermo Fisher Scientific | A11004 | Waltham, USA |
| anti-rabbit IgG  Alexa Fluor-488 | goat | Thermo Fisher Scientific | A11008 | Waltham, USA |
| anti-rabbit IgG  Alexa Fluor-555 | goat | Thermo Fisher Scientific | A21428 | Waltham, USA |
| anti-rabbit IgG  Alexa Fluor-647 | goat | Thermo Fisher Scientific | A21244 | Waltham, USA |
| anti-rat IgG  Alexa Fluor-555 | goat | Thermo Fisher Scientific | A21434 | Waltham, USA |

Supplementary file 1d: Fluorescent probe.

| **Antibody (clone)** | **Dilution** | **Manufacturer** | **Catalogue number** | **Registered office** |
| --- | --- | --- | --- | --- |
| 4,6-diamidino-2-phenylindole  (DAPI) | 300 nM | Biolegend | 422801 | San Diego, USA |

Supplementary file 1e: Primary antibodies used for Western blotting (WB).

| **Antibody (clone)** | **Host species** | **Dilution** | **Manufacturer** | **Catalogue number** | **Registered office** |
| --- | --- | --- | --- | --- | --- |
| Actin (8H10D10) | mouse | 1:20,000 | Cell Signaling Technologies | 3700S | Danvers, USA |
| Actin (13E5) | rabbit | 1:20,000 | Cell Signaling Technologies | 4970S | Danvers, USA |
| AKT (pan) (C67E7) | rabbit | 1:2500 | Cell Signaling Technologies | 4691S | Danvers, USA |
| BDNF | rabbit | 1:500 | Elabscience | 18244.200 | Houston, USA |
| pAKT (S473) D9E  XP® | rabbit | 1:2500 | Cell Signaling Technologies | 4060S | Danvers, USA |
| pTrkB | mouse | 1:500 | Santa Cruz | SC-8058 | Santa Cruz, USA |

Supplementary file 1f: IR-dye conjugated secondary antibodies used for WB.

| **Antibody (clone)** | **Host species** | **Manufacturer** | **Catalogue number** | **Registered office** |
| --- | --- | --- | --- | --- |
| Anti-mouse DyLight™ 680 | goat | Cell Signaling Technologies | 5470S | Danvers, USA |
| Anti-mouse DyLight™ 800 | goat | Cell Signaling Technologies | 5257S | Danvers, USA |
| Anti-rabbit DyLight™ 680 | goat | Cell Signaling Technologies | 5366S | Danvers, USA |
| Anti-rabbit DyLight™ 800 | goat | Cell Signaling Technologies | 5151S | Danvers, USA |

Supplementary file 1g: Primers for RT-PCR.

| **Primer name** | **Sequence (5’->3’)** | **Size**  **base pair (bp)** |
| --- | --- | --- |
| HTR2A-For | TTGGGCTACAGGACGATT | 383 |
| HTR2A-Rev | GAAGAAAGGGCACCACATC |  |
| 18s rRNA-For | AAACGGCTACCACATCCAAG | 143 |
| 18s rRNA-Rev | CCTCCAATGGATCCTCGTTA |  |
| ANK3-For | TGGAATGATTGAACGGAGTACAGG | 142 |
| ANK3-Rev | AGTGAAGCCTGACTTGGCTG |  |
| ARC-For | TCAGCTCCAGTGATTCACGC | 163 |
| ARC-Rev | GGGAACCTTGAGACCTGTTGT |  |
| BDNF-For | TTTGGTTGCATGAAGGCTGC | 199 |
| BDNF-Rev | TGAGGACCAGAAAGTTCGGC |  |
| BCL11B-For | CAACCCGCAGCACTTGTC | 80 |
| BCL11B-Rev | CCTCGTCTTCTTCGAGGATGG |  |
| DLG4-For | AGCCCCAGGATATGAGTTGC | 90 |
| DLG4-Rev | CCCAGACCTGAGTTACCCCT |  |
| FOS-For | CAAGCGGAGACAGACCAACT | 177 |
| FOS-Rev | AGTCAGATCAAGGGAAGCCA |  |
| GAD1-For | ATCCTGGTTGACTGCAGAGAC | 80 |
| GAD1-Rev | CCAGTGGAGAGCTGGTTGAA |  |
| GRIA2-For | GGATCCTCATTAAGAACCCCAGT | 148 |
| GRIA2-Rev | TGAGGGCACTGGTCTTTTCC |  |
| GRIA4-For | AGGTGAATGTGGACCCAAGG | 177 |
| GRIA4-Rev | AAGGTCAGCTTCATTCTCTTCG |  |
| GRIN1-For | GGCAACACCAACATCTGGAA | 71 |
| GRIN1-Rev | CCATCCGCATACTTGGAAGAC |  |
| GRIN2B-For | CTCACCCCCTTTCCGCTTT | 156 |
| GRIN2B-Rev | AAGGCATCCAGTTTCCCTGTT |  |
| NEUROD6-For | ACTCAGCCTGAAAAGATTTGG | 89 |
| NEUROD6-Rev | TGGTTCTCTAATCTTAAATTACCTT |  |
| NPAS4-For | AACACTACCGCCTGTTGGC | 133 |
| NPAS4-Rev | GCAGTAATGGGTCCCTCTGG |  |
| NRXN1-For | GTGTTTTGCCGGTGCTGTTA | 133 |
| NRXN1-Rev | CTTTTACCTTGGTCGCCCATC |  |
| RBFOX3-For | GCGCTGAGCCCGTTGAAAT | 104 |
| RBFOX3-Rev | CTCCTTCTGGACCGTCCTTG |  |
| Mycoplasma-For | GGGAGCAAACAGGATTAGATACCCT | > 200 |
| Mycoplasma-Rev | TGCACCATCTGTCACTCTGTTAACCTC |  |
| SLC17A7-For | AGCTGGGATCCAGAGACTGT | 116 |
| SLC17A7-Rev | CCGAAAACTCTGTTGGCTGC |  |
| SLC17A6-For | TCAGATTCCGGGAGGCTACA | 175 |
| SLC17A6-Rev | TGGGTAGGTCACACCCTCAA |  |
| SV2A-For | AACCTAGACCAGGCACTCAT | 99 |
| SV2A-Rev | ACCCCTCCCCACAGTTACTTA |  |
| SYN1-For | CAGCTCAACAAATCCCAGTCTC | 99 |
| SYN-1Rev | GGTCTCAGCTTTCACCTCGT |  |
| TBR1-For | ACGAACAACAAAGGAGCTTCA | 68 |
| TBR1-Rev | TGGTACTTGTGCAAGGACTGTA |  |
| TUBB3-For | GAGCGGATCAGCGTCTACTA | 77 |
| TUBB3-Rev | GGTTCCAGGTCCACCAGAA |  |
